# The Mental Health of Patients With Psychotic Disorder From a Positive, Multidimensional and Recovery Perspective

**DOI:** 10.3389/fpsyg.2022.857598

**Published:** 2022-07-04

**Authors:** Miriam Broncano-Bolzoni, Mònica González-Carrasco, Dolors Juvinyà-Canal, MTeresa Lluch-Canut

**Affiliations:** ^1^Institut d'Assistència Sanitària, Girona, Spain; ^2^Department of Nursing, University of Girona, Girona, Spain; ^3^Quality of Life Research Institute, University of Girona, Girona, Spain; ^4^Research Group Health and Healthcare, University of Girona, Girona, Spain; ^5^Mental Health Sciences Department, School of Nursing, University of Barcelona, Barcelona, Spain

**Keywords:** positive mental health, recovery, subjective wellbeing, insight, psychotic disorder

## Abstract

Positive mental health (PMH) and mental illness are distinct, yet interrelated, constructs. However, this relationship has yet to be adequately established. We aimed to evaluate the level of PMH and its relationship with sociodemographic and clinical determinants as well as to explore the relationship between PMH and the positive constructs of recovery, subjective wellbeing (SWB), insight and functioning in patients with psychotic disorder. A multicenter, descriptive, cross-sectional and correlational study with a sample of 347 patients with psychotic disorder was conducted. The following assessment instruments were used: Positive Mental Health Questionnaire, Maryland Assessment of Recovery in Serious Mental Illness scale, Insight Scale, Personal Wellbeing Index-Adult version (PWI-A), Overall Life Satisfaction (OLS) and Global Assessment of Functioning scale. The mean global level of PMH was 116.16 (range of 39–156, SD = 19.39). Significant differences were found in PMH in relation to sociodemographic (sex, civil status and employment situation) and clinical variables (family history of mental disorders, number of prescribed antipsychotics, treatment with anxiolytics, treatment with antidepressants and suicide attempts). PMH was significantly and positively correlated with recovery (*r* = 0.760), SWB (PWI-A: *r* = 0.728 and OLS: *r* = 0.602) and functioning (*r* = 0.243), and negatively with insight (*r* = −0.335). These results can lead to a major change in mental health care. If actions are taken to increase PMH, then recovery, SWB and functioning will also increase. At the same time, interventions should be carried out to boost insight, since increasing PMH could decrease insight, all resulting in better quality of life for patients with psychotic disorder.

## Introduction

According to the World Health Organization (2005), mental health is a “state of wellbeing in which the individual realizes his or her own abilities, can cope with the normal stresses of life, can work productively and fruitfully, and is able to make a contribution to his or her community.” This definition has its foundation in positive mental health (PMH), a construct first proposed by Jahoda (1958), who described PMH as individuals' attitudes toward themselves and the environment and their ability to adapt to situations.

Mental health and mental illness are traditionally conceptualized as being on opposite ends of the same continuum. However, evidence indicates that they represent two distinct, yet interrelated, dimensions. Therefore, the absence of the disorder does not guarantee the presence of health, and *vice versa* (Keyes, 2013). Based on this belief, people with mental disorders can experience at the same time positive emotions, form close relationships, have a purposeful life and function well by relating to PMH. This means that a patient suffering from a mental disorder will require not only a clinical assessment of the disease process, but also special care from a positive perspective. The positive aspect of mental health is currently considered in mental health care systems (e.g., Forsman et al., 2015; Wahlbeck, 2015).

In this respect, the PMH construct has been studied for years based on Lluch-Canut's Multifactor PMH model (1999), made up of six interrelated factors: F1-Personal Satisfaction (F1), F2-Prosocial Attitude (F2), F3-Self-Control (F3), F4-Autonomy (F4), F5-Problem-Solving and Self-Actualization (F5), and F6-Interpersonal Relationship Skills (F6). To evaluate this multifactorial model, the author created the Positive Mental Health Questionnaire (PMHQ), which is described in detail in the Methods section. Lluch-Canut's PMHQ (1999) has been used in various studies and settings: in patients with schizophrenia (Miguel, 2014); in chronic patients (Lluch-Canut et al., 2013; Puig-Llobet et al., 2020); in carers of patients with schizophrenia (Albacar, 2014), and in university students (Roldán-Merino et al., 2017; Sequeira et al., 2020). The questionnaire has also been translated into Portuguese (Sequeira et al., 2014) and Turkish (Teke and Baysan Arabaci, 2018). Although there is increasingly more research on PMH, it has been little researched in people with psychotic disorder (Miguel, 2014; Jeyagurunathan et al., 2017; Na and Lim, 2020), and only one study (Miguel, 2014) uses the PMHQ. It is therefore important to assess PMH and its factors in people with mental illness, as it will help health professionals in better understanding these individuals' specific needs and also in implementing both health promotion and prevention activities to improve their mental health.

Following this line of research, we set out to study PMH supplemented with other positive constructs, such as recovery, subjective wellbeing (SWB), insight and functioning, and in this way characterize the relationship between them. We briefly describe below some highlights in the scientific literature on the use of these constructs.

Recovery research focusing on people with mental disorders is still relatively recent. However, in the last few years, the concept of recovery has experienced significant momentum both at clinical and policy level, being incorporated into health policies in various countries (e.g., Burgess et al., 2011). The Substance Abuse Mental Health Services Administration (2005) defined mental health recovery as “a journey of healing and transformation, enabling a person with a mental health problem to live a meaningful life in a community of his or her choice while striving to achieve his or her full potential” (p. 1), this being one of the most widely used and accepted definitions.

SWB can be defined as a normally positive state of mind that involves the whole life experience. One of the models proposed to explain how SWB works is the Homeostatic Theory and stems from the idea that SWB, under normal conditions, is managed by a psychological, homeostatic system in such a way that people are usually satisfied with their life and this does not tend to change substantially over time (Cummins, 2010).

In recent decades, the term “insight” has aroused great interest in the field of mental health due to the relationship it has been shown to have with numerous variables, which have an important impact on mental disorders. Insight refers to the degree of awareness that the individual has of their disease. It is considered a multidimensional construct that affects different areas with different intensity and may be present in some areas, but not in others (David, 1990).

Functioning is the individual's ability to effectively carry out the activities of daily living (World Health Organization, 2001). By definition, psychotic disorders involve suffering and impaired functioning (American Psychiatric Association, 2013).

Evidence exists of a link between recovery, SWB, insight and functioning and mental disorders (Cannavò et al., 2016; Chan et al., 2018; Widschwendter et al., 2018; Yu et al., 2020). However, no previous studies have been known to address the innovative PMH construct and its correlation with this set of positive measures. It is important to manage as many positive measures as possible in order to develop multi-component intervention programs. This is the reason why we have conducted this study. Its objectives are to evaluate the level of PMH and its relationship with sociodemographic and clinical determinants as well as to explore the relationship between PMH and the positive constructs of recovery, SWB, insight and functioning in patients with psychotic disorder being treated in mental healthcare facilities in Catalonia (Spain).

Although this is a descriptive and exploratory study, the main hypothesis that has guided the analysis of the data collected is defined as follows. Specifically, the level of PMH in people with psychotic disorder is thought to correlate positively with recovery, SWB and functioning, and negatively with insight.

## Materials and Methods

### Design

Multicentre, cross-sectional, descriptive, correlational study.

### Participants

The sample was made up of 347 patients diagnosed with a psychotic disorder from four healthcare institutions in Catalonia (Spain): the Healthcare Institute (IAS), the Mar Health Park, the Benito Menni Mental Healthcare Complex, and the Sant Joan de Déu Health Park. The sample size calculations were based on the expectation of finding a clinically relevant medium-effect size in primary outcomes with a two-sample t-test, while also taking into consideration a three- or four-group design. A minimum sample size of 200 would be needed for a significance level of α = 0.05 (two-sided), power of 1–β = 80% and an expected attrition rate of 10%. Moreover, with this sample size, a relevant simple correlation (*r* ≥ 0.25) can be detected. Subjects were selected by non-probability sampling between February 2015 and May 2016, using the following inclusion criteria: adults with a DSM-5 (American Psychiatric Association, 2013) diagnosis of psychotic disorder; patients in the stabilization or recovery phase of psychotic disorder, and with knowledge of Spanish or Catalan. Exclusion criteria included: patients diagnosed with intellectual disability or any type of organic mental disorder, such as dementia (DSM-5); patients diagnosed with substance- or medication-induced psychotic disorder, psychotic disorder due to other medical conditions, delusional disorder, schizophreniform disorder or schizoaffective disorder (DSM-5), and patients in an acute phase at the time of assessment.

### Measures

The study variables were the following: (a) sociodemographic variables, (b) clinical variables, (c) PMH, (d) recovery, (e) SWB, (f) insight, and (g) functioning. The instruments used are described in the following sections.

#### Positive Mental Health Questionnaire

PMH was assessed using the Positive Mental Health Questionnaire (PMHQ) (Lluch-Canut, 1999). This questionnaire is composed of 39 items, distributed among 6 factors that make up the PMH Multifactor Model: F1-Personal Satisfaction, F2-Prosocial Attitude, F3-Self-Control, F4-Autonomy, F5-Problem-Solving and Self-Actualization, and F6-Interpersonal Relationship Skills. The items have been formulated as (positive or negative) statements and the response to each item is assessed on a 4-point scale on frequency: “always or almost always,” “quite often,” “sometimes,” and “never or almost never.” The scores enable us to obtain a PHM value as a single measure (including all the items in the questionnaire) and also specific values for each factor. The psychometric analyses of the original PHMQ conducted with a sample of nursing students were favorable (Lluch-Canut, 1999). In this study, Cronbach's α on the global scale was 0.93, and by factors: F1 = 0.85; F2 = 0.60; F3 = 0.82; F4 = 0.78; F5 = 0.83; F6 = 0.78.

#### Maryland Assessment of Recovery in Serious Mental Illness

To evaluate the recovery variable, the Maryland Assessment of Recovery in Serious Mental Illness (MARS) (Drapalski et al., 2012) was used. The MARS is a self-administered instrument made up of 25 items and uses a 5-point Likert-type response scale: “not at all,” “a little,” “somewhat,” “quite a lot,” and “a lot.” This measure was developed based on the SAMHSA definition of recovery and assesses six domains: empowerment, holistic recovery, non-linear recovery, strengths-based recovery, a sense of responsibility, and hope. The instrument provides an overall score (the sum of scores for all items) of the person's ability to recover, ranging from 25 (low) to 156 points (high). The psychometric analyses of the MARS were favorable (Drapalski et al., 2012, 2016). The Spanish and Catalan versions was validated for this study, obtaining favorable psychometric results, both in terms of reliability [Spanish version: Cronbach's α = 0.96; interclass correlation coefficient (ICC) = 0.96; Catalan version: Cronbach's α = 0.95; ICC = 0.95] and (convergent and construct) validity. Convergent validity showed a high correlation between the MARS and the PMHQ (*r* = 0.76), the PWI-A (*r* = 0.70) and the OLS (*r* = 0.65). Construct validity showed a good fit for a single-factor model.

#### Personal Wellbeing Index-Adult Version

SWB was evaluated using the Personal Wellbeing Index-Adult Version (PWI-A) (Cummins et al., 2003; International Wellbeing Group, 2013). The PWI-A is a 7-item, self-administered scale that measures satisfaction with different life domains: standard of living, health, life achievements, personal relationships, personal safety, community connectedness, and future security. The items are rated on a scale ranging from 0 (“no satisfaction at all”) to 10 (“completely satisfied”). The final score is obtained by calculating the mean score of the items and converting them to a 0–100-point scale format. The original version of the domains included in the PWI-A and their back translation into Spanish and Catalan were carried out by Casas et al. (2008). As a result of this adaptation, the community connectedness domain was replaced by satisfaction with the groups the respondent belongs to. The normative range for Western populations is 70 to 80 points. The authors have assured the instrument has good psychometric properties (International Wellbeing Group, 2013). In this study, Cronbach's α was 0.88.

#### Overall Life Satisfaction

The Overall Life Satisfaction (OLS) (Campbell et al., 1976) was also used to evaluate SWB. This is a single-item scale that evaluates overall life satisfaction on a scale ranging from 0 (extremely dissatisfied) to 10 (extremely satisfied). The formulation in the most common Spanish version (Casas, 2010, p. 92) is: Overall, how satisfied are you with your life these days?”. Campbell et al. (1976) suggested supplementing OLS with scales for evaluating specific life domains. The IWG also recommends including OLS to evaluate overall life satisfaction, even though it is not part of the PWI-A (International Wellbeing Group, 2013).

#### Insight Scale

The Insight Scale (IS) (Birchwood et al., 1994) was used to measure insight. This is a self-administered scale that assesses insight from a multi-dimensional perspective. It is composed of 8 items that assess three domains: D1-awareness of having symptoms (D1), D2-awareness of having a mental illness (D2), and D3-awareness of the need for treatment (D3). The scale provides an overall insight score as well as specific values for the three dimensions. The maximum score is 12 points (high level of insight) and the minimum score is 0 (lack of insight). A score of 9 points or above is considered adequate insight. The instrument was validated in Spanish by Camprubi et al. (2008) and displayed satisfactory psychometric properties. In this study, Cronbach's α on the global scale was 0.84, and by domains: D1 = 0.67, D2 = 0.61 and D3 = 0.85.

#### Global Assessment of Functioning Scale

The GAF (American Psychiatric Association, 2002) was used to assess functioning. This instrument provides a multiaxial assessment of a person's global functioning. It consists of a single-item rated on a scale ranging from 100 (superior functioning in a wide range of activities) to 1 (persistent danger of severely hurting self or others or persistent inability to maintain minimal personal hygiene or serious suicidal act with clear expectation of death). This scale was developed for clinical use and was also incorporated as Axis 5 in versions III-TR, IV and IV-TR of the DSM (American Psychiatric Association, 2002). This scale has been used in diverse populations, regardless of their diagnosis. It has good psychometric properties.

### Procedure

Once the study protocol had been approved by the Clinical Research Ethical Committee of the Healthcare Institute of Girona, the Benito Menni Mental Healthcare Complex, the Mar Health Park and the research committee of the Sant Joan de Déu Health park, data collection began. Subjects were selected using the inclusion and exclusion criteria described above, in consultation with the referring psychiatrist to corroborate the state of stabilization of the mental disorder. The researchers responsible for the study informed the participants about the study by giving them a “User Information Sheet,” which contained the relevant information necessary for the patient to decide whether or not to participate in the study. Prior to data collection, the researchers responsible for the study clarified all doubts with the participants and allowed them to ask any questions they considered appropriate, and, in accordance with the regulations in force, obtained the subject's written informed consent. Once the informed consent had been signed by the participant, data collection for the different instruments began. First, the researcher collected the sociodemographic and clinical variables, then the participant self-completed the following instruments: the PMHQ, the PWI-A, the OLS, the MARS and the IS. The GAF was provided by the referring psychiatrist. The approximate duration of data collection was 30–45 mins.

### Statistical Analysis

Descriptive analysis based on central tendency and dispersion measures was conducted for quantitative variables, and on absolute and relative frequencies for categorical variables. The Pearson correlation coefficient between two quantitative variables was calculated. The relationships between a quantitative and a qualitative variable were analyzed using the Student's t test or a factor analysis of variance (ANOVA), depending on the response modalities of each variable. Finally, a multiple linear regression analysis was performed using the explanatory variables (recovery, SWB including satisfaction with specific life domains and overall life satisfaction, insight, functioning and socio-demographic and clinical variables) on the response variable (global PMH). The confidence level was taken as 95% and the difference between variables was considered significant when the level of statistical significance was less than, or equal to 0.05. For descriptive, univariate and bivariate analyses, IBM SPSS version 25 was used. Finally, multivariate analysis was carried out with STATA, version 13.1.

## Results

### Subjects' Characteristics

It was observed that the mean age of the 347 patients that made up the sample was 41.29 years, the majority were men (66.9%), single (75.2%), with a primary or secondary school level of education (81.2%), disabled (57.1%), and living with someone (81.0%). Regarding the clinical variables of the sample under study, the mean number of years of evolution of the psychotic disorder was 12.57 years, 47.3% had a family history of mental disorders, the mean number of hospital admissions since the onset of the disorder was 3.37, 46.1% had a history of organic pathologies, with a higher prevalence of patients taking oral medication (49.2%), 35.4% taking anxiolytics and 28.5% on antidepressants, and 24.8% had attempted suicide (Table 1).

**Table 1:** Sociodemographic and clinical characteristics, PMH on a global level and by factors, recovery, SWB, insight and functioning (*N* = 347).

**Variable**	**Mean (SD)**	***N* (%)**
**Age**	41.29 (11.29)	
**Sex**		
Man		232 (66.9)
Woman		114 (33.1)
**Civil status**		
Single		261 (75.2)
Married		47 (13.6)
Separated/ divorced/ widowed		39 (11.2)
**Level of education**		
No formal education / primary education not completed		28 (8.1)
Primary education		148 (42.6)
Secondary education		134 (38.6)
Higher education		37 (10.7)
**Employment situation**		
Active worker		39 (11.2)
Inactive worker		48 (13.8)
Disabled		198 (57.1)
Others		62 (17.9)
**People they live with**		
Alone		66 (19.0)
With others		281 (81.0)
**Evolution of the psychotic disorder in years**	12.57 (10.17)	
**Family history of mental disorders**		
Yes		164 (47.3)
No		183 (52.7)
**Number of hospital admissions**	3.37 (3.79)	
**Medical history**		
Yes		164 (47.3)
No		183 (52.7)
**Number of prescribed antipsychotics**		
1 antipsychotic		190 (54.7)
2 antipsychotics		120 (34.6)
3 or more antipsychotics		37 (10.7)
**Treatment with anxiolytics**		
Yes		123 (35.4)
No		224 (64.6)
**Treatment with antidepressants**		
Yes		99 (28.5)
No		248 (71.5)
**Suicide attempts**		
Yes		86 (24.8)
No		261 (75.2)
**Overall PMH**	116.16 (19.39)	
F1- Personal satisfaction	24.95 (5.18)	
F2- Prosocial behavior	15.66 (2.78)	
F3-Self-control	14.55 (3.51)	
F4-Autonomy	14.36 (3.60)	
F5-Problem-solving and self-actualization	27.44 (5.28)	
F6-Interpersonal relationship skills	19.20 (4.46)	
**Recovery**	91.63 (19.26)	
**SWB**		
PWI-A	60.20 (19.11)	
OLS	6.41 (2.91)	
**Total insight**	8.83 (3.06)	
D1-Awareness of symptoms	2.99 (1.32)	
D2-Awareness of illness	2.45 (1.28)	
D3-Need for treatment	3.39 (1.07)	
**Functioning**	61.46 (12.30)	

*M, mean; OLS, overall life satisfaction; PMH, positive mental health; PWI-A, Personal Wellbeing Index-Adult; SD, standard deviation; SWB, subjective wellbeing*.

As for positive health measures, the mean global level of PMH was 116.16, with a minimum value of 62 and a maximum of 154. The level of recovery was 91.63, with a minimum value of 43 and a maximum of 125. The mean PWI-A score was 60.20, and 6.41 for the OLS. The patients' mean insight score was 8.83. Forty-five-point three percent of the subjects presented adequate insight (≥9 points). Finally, the mean level of functioning was 61.46, with a minimum value of 35 and a maximum of 95 (Table 1).

### Relationship Between Level of PMH and Sociodemographic and Clinical Variables

Table 2 shows the relationship between PMH and the sociodemographic and clinical variables. Statistically significant differences were found in two factors regarding sex: F2 (*p* < 0.001) and F6 (*p* = 0.001). Women reported having greater prosocial behavior as well as more interpersonal relationship skills.

**Table 2:** Relationship between global PMH and by factors and sociodemographic and clinical variables.

**Variable**	**F1-Personal**		**F2-Prosocial**		**F3-Self-control**		**F4-Autonomy**		**F5-Problem-solving**		**F6-Interpersonal**		**Global PMH**	
	**satisfaction**		**attitude**				**and self-actualization**		**relationship skills**		
	**M (SD)**	**t/F/r**	**M (SD)**	**t/F/r**	**M (SD)**	**t/F/r**	**M (SD)**	**t/F/r**	**M (SD)**	**t/F/r**	**M (SD)**	**t/F/r**	**M (SD)**	**t/F/r**
**Age**		0.01		0.03		0.09		0.09		−0.06		0.02		0.03
**Sex**														
Man	25.00 (5.09)	0.26	15.29 (2.72)	−3.62***	14.65 (3.47)	0.81	14.24 (3.47)	−0.88	24.27 (5.18)	−0.86	18.65 (4.47)	−3.31**	115.10 (19.36)	−1.45
Woman	24.85 (5.38)		16.42 (2.75)		14.33 (3.60)		14.60 (3.84)		27.79 (5.47)		20.30 (4.24)		118.10 (19.36)	
**Civil status**														
Single	24.78 (5.18)	0.78	15.27 (2.80)	10.84***	14.46 (3.50)	0.30	14.24 (3.54)	4.22*	27.00 (5.23)	3.78*	18.85 (4.40)	3.23*	114.62 (19.38)	3.37*
Married	25.81 (4.86)		17.02 (2.31)		14.79 (3.55)		14.74 (3.81)		28.81 (5.36)		20.34 (4.44)		120.51 (17.75)	
Separated / divorced / widowed	25.05 (5.60)		16.59 (2.46)		14.82 (3.57)		15.85 (3.44)		28.77 (5.14)		20.13 (4.62)		121.20 (20.15)	
**Level of education**														
No formal education	24.75 (6.08)	0.66	15.93 (3.25)	0.74	14.04 (4.11)	0.30	14.32 (4.06)	0.40	28.29 (4.21)	1.88	18.43 (5.34)	0.87	115.75 (19.73)	0.88
Primary education	24.57 (5.15)		15.48 (2.71)		14.49 (3.41)		14.16 (3.54)		26.68 (5.37)		18.93 (4.22)		114.31 (18.35)	
Secondary education	25.43 (4.97)		15.64 (2.80)		14.69 (3.49)		14.62 (3.52)		28.04 (5.17)		19.46 (4.39)		117.90 (19.80)	
Higher education	24.95 (5.18)		16.29 (2.60)		14.68 (3.57)		14.24 (3.83)		27.68 (5.80)		19.86 (4.92)		117.59 (21.71)	
**Employment situation**														
Active worker	26.44 (4.71)	1.63	16.85 (2.68)	5.04**	15.67 (3.09)	2.15	14.54 (3.73)	0.82	30.15 (4.42)	4.90**	21.26 (4.48)	3.80*	124.90 (17.85)	4.17**
Inactive worker	25.06 (5.47)		15.65 (2.77)		14.62 (3.25)		14.87 (3.68)		27.81 (5.01)		18.65 (4.42)		116.67 (18.30)	
Disabled	24.52 (5.29)		15.24 (2.69)		14.21 (3.56)		14.11 (3.51)		26.74 (5.31)		18.80 (4.36)		113.62 (19.10)	
Others	25.31 (4.80)		16.27 (2.87)		14.87 (3.67)		14.64 (3.75)		27.71 (5.38)		19.60 (4.48)		118.40 (20.55)	
**People they live with**														
Alone	25.21 (4.79)	0.45	15.94 (2.62)	0.90	15.26 (2.97)	1.83	15.30 (3.58)	2.39*	27.94 (4.84)	0.85	19.65 (4.14)	0.92	119.30 (17.42)	1.46
With others	24.89 (5.28)		15.60 (2.82)		14.38 (3.61)		14.13 (3.57)		27.33 (5.38)		19.09 (4.53)		115.42 (19.78)	
**Evolution of psychotic disorder in years**		−0.01		−0.05		−0.03		−0.05		−0.11*		−0.05		−0.05
**Family history of mental disorders**														
Yes	24.38 (5.47)	−1.95	15.47 (2.98)	−1.23	13.85 (3.69)	3.55***	14.10 (3.80)	1.24	26.86 (5.56)	1.94	19.12 (4.89)	−0.29	113.80 (21.13)	2.16*
No	25.46 (4.87)		15.84 (2.58)		15.17 (3.22)		14.58 (3.40)		27.96 (4.97)		19.26 (4.04)		118.28 (17.47)	
**Number of hospital admissions**		0.08		0.00		0.05		0.02		0.01		0.01		0.04
**Medical history**														
Yes	24.58 (5.28)	−1.24	15.69 (2.80)	0.19	14.39 (3.55)	−0.79	14.47 (3.80)	0.53	27.19 (5.11)	−0.84	19.18 (4.42)	−0.06	115.50 (18.67)	−0.59
No	25.27 (5.09)		15.64 (2.77)		14.68 (3.48)		14.26 (3.43)		27.66 (5.42)		19.21 (4.50)		116.73 (20.02)	
**Number of prescribed antipsychotics**														
1 antipsychotic	25.14 (5.06)	11.91***	15.89 (2.70)	11.60	14.74 (3.49)	6.28**	14.50 (3.69)	4.83**	27.63 (5.47)	4.85**	19.65 (4.21)	3.86*	117.55 (20.05)	8.34***
2 antipsychotics	25.80 (4.55)		15.47 (2.86)		14.83 (3.21)		14.65 (3.34)		27.92 (4.64)		19.01 (4.53)		117.65 (16.45)	
≥3 antipsychotics	21.24 (6.22)		15.13 (2.86)		12.65 (4.01)		14.65 (3.56)		24.95 (5.69)		17.49 (5.09)		104.11 (20.97)	
**Treatment with anxiolytics**		−1.72		−1.27		−2.13*		−2.23*		−2.91**		−2.08*		−2.72**
Yes	24.31 (5.58)		15.41 (2.86)		14.01 (3.38)		13.78 (3.80)		26.34 (5.54)		18.53 (4.57)		112.37 (20.13)	
No	25.31 (4.93)		15.80 (2.73)		14.84 (3.55)		14.67 (3.45)		28.05 (5.04)		19.56 (4.36)		118.24 (18.69)	
**Treatment with antidepressants**														
Yes	23.50 (5.63)	−3.34**	15.82 (2.74)	0.66	13.62 (3.51)	−3.16**	13.38 (3.69)	−3.23**	26.10 (5.37)	−3.03**	18.39 (4.50)	−2.13*	110.82 (20.20)	−3.29**
No	25.53 (4.88)		15.60 (2.80)		14.92 (3.44)		14.75 (3.49)		27.98 (5.15)		19.52 (4.41)		118.29 (18.67)	
**Suicide attempts**														
Yes	22.93 (5.89)	−3.84***	15.23 (2.75)	−1.66	13.30 (3.26)	−3.87***	13.46 (3.90)	−2.68**	25.73 (5.33)	−3.52***	18.57 (4.76)	−1.50	109.23 (20.45)	−3.90***
No	25.62 (4.75)		15.80 (2.78)		14.96 (3.49)		14.65 (3.45)		28.01 (5.15)		19.40 (4.34)		118.44 (18.51)	

*F, one-way ANOVA; M, mean; PMH, positive mental health, SD, standard deviation; SWB, subjective wellbeing; t, independent t test+*.

*p < 0.05.

**p < 0.01.

***p < 0.001.

Significant differences were observed in four factors regarding marital status: F2 (*p* < 0.001), F4 (*p* = 0.015), F5 (*p* = 0.024) and F6 (*p* = 0.041), and at a global level (*p* = 0.036). Separated, divorced and widowed subjects had greater overall PMH and autonomy. On the other hand, subjects that were married reported greater prosocial behavior, problem-solving and self-actualization, and interpersonal relationship skills. In contrast, single men and women had the lowest scores.

Statistically significant differences were observed between employment status and three factors: F2 (*p* = 0.002), F5 (*p* = 0.002) and F6 (*p* = 0.010) and overall PMH (*p* = 0.006). Specifically, active workers had the highest scores. On the contrary, patients with some type of disability had lower scores in prosocial behavior, problem-solving and self-actualization and global PMH. Inactive workers reported lower interpersonal relationship skills.

Regarding the people they lived with, there was a significant correlation in relation to F4 (*p* = 0.017). Subjects who lived alone showed greater autonomy. No significant differences were detected between the level of PMH and other sociodemographic variables.

On the other hand, a negative and significant, but low, relationship was found between the years of evolution of the psychotic disorder and factor F5 (*p* = 0.049).

There was a significant correlation in factor F3 (*p* < 0.001) and in global PMH (*p* = 0.031). Subjects with a family history of mental disorders showed a lower capacity for self-control and lower global PMH.

Significant differences were found between prescribed antipsychotics and the global level of PMH in all the factors it is made up of, except F2. Specifically, patients on three or more antipsychotics had lower scores.

Significant differences were found regarding treatment with anxiolytics in four factors: F3, F4, F5 and F6 and in global PMH. Patients taking prescribed anxiolytics had a lower score.

Statistically significant differences were observed in treatment with antidepressants and global PMH and in all the factors it is made up of, except F2. Patients being treated with antidepressants had lower scores.

Finally, statistically significant differences were found between suicide attempts, the global level of PMH and four factors: F1 (*p* < 0.001), F3 (*p* < 0.001), F4 (*p* = 0.008), F5 (*p* < 0.001). Patients who had attempted suicide had lower scores. No significant differences were identified between the level of PMH and other clinical variables.

### Relationship Between Levels of PMH and Recovery, SWB, Insight and Functioning

A strong and significant correlation was obtained between the PMH constructs and recovery (*r* = 0.760). In the positive sense, the correlation indicated that the higher the global level of PMH, the greater the capacity for recovery. Table 3 shows that all the PMH factors correlated positively and significantly with recovery, ranging from 0.439 (F2) to the highest correlation, 0.716 (F5). Strong and significant correlations were obtained between PMH and SWB, evaluated *via* the PWI-A and the OLS; overall, they were 0.728 and 0.602, respectively. The correlation between global PMH and insight was low, but significant (*r* = −0.335). In the negative sense, the correlation indicated that the higher the global level of PMH, the lower the sense of insight. When analyzing the relationship between the specific PMHQ factors and the three dimensions of IS, it was observed that all the factors/dimensions correlated negatively and significantly. Finally, a low but significant correlation was obtained between PMH and functioning (*r* = 0.243).

**Table 3:** Pearson's correlation between global PMH and by factors, recovery, SWB, insight and functioning.

	**F1-Personal** **satisfaction**	**F2-Prosocial** **attitude**	**F3-Self-control**	**F4-Autonomy**	**F5-Problem-solving** **and self-actualization**	**F6-Interpersonal** **relationship skills**	**Global PMH**
**Recovery**	0.709**	0.439**	0.496**	0.489**	0.716**	0.574**	0.760**
**SWB**							
**PWI-A**	0.724**	0.429**	0.509**	0.463**	0.604**	0.570**	0.728**
**OLS**	0.637**	0.302**	0.424**	0.374**	0.541**	0.415**	0.602**
**Total insight**	−0.311**	−0.194**	−0.274**	−0.242**	−0.258**	−0.259**	−0.335**
D1-Awareness of symptoms	−0.171**	−0.117*	−0.191**	−0.136*	−0.160**	−0.145**	−0.199**
D2-Awareness of illness	−0.394**	−0.232**	−0.310**	−0.293**	−0.283**	−0.318**	−0.399**
D3-Need for treatment	−0.206**	−0.132*	−0.177**	−0.171**	−0.200**	−0.180**	−0.234**
**Functioning**	0.221**	0.236**	0.168**	0.133*	0.180**	0.201**	0.243**

*OLS, overall life satisfaction; PMH, positive mental health; PWI-A, Personal Wellbeing Index-Adult; SWB, subjective wellbeing*.

^*^p < 0.05.

^**^p < 0.01.

### Multivariate Analysis of Factors Associated With Positive Mental Health

The results of the multiple linear regression analysis are presented in Table 4. It can be observed that recovery (β = 0.47; *p* < 0.001), SWB [PWI-A: (β = 0.38; *p* < 0.001)], functioning (β = 0.11; *p* = 0.45), age (β = 0.16; *p* = 0.04) and being separated, divorced or widowed (β = 5.18; *p* = 0.008) showed a statistically significant and positive association with global PMH. In contrast, insight (β = −0.46; *p* = 0.031) and having a family history of mental disorder (β = −2.63; *p* = 0.029) showed a statistically significant and negative association with global PMH.

**Table 4:** Multivariate regression analysis of factors associated with PMH.

	**Crude model**			**Adjusted model**		
	**β**	**SD**	**95% CI**	**β**	**SD**	**95% CI**
**Recovery**	0.76	0.04	0.70–0.83***	0.47	0.05	0.37–0.56***
**SWB**						
PWI-A	0.74	0.04	0.67–0.81***	0.38	0.05	0.29–0.47***
OLS	5.10	0.36	4.38–5.81***			
**Total insight**	−2.12	0.32	−2.75 to −1.49***	−0.46	0.21	−0.87 to −0.04*
**Functioning**	0.38	0.08	0.22–0.55***	0.11	0.53	0.003–0.21*
**Age**	0.05	0.09	−0.13 to 0.22	0.16	0.06	0.05–0.27**
**Sex**						
Man	Ref					
Woman	3.19	2.21	−1.15 to 7.53			
**Civil status**						
Single	Ref			Ref		
Married	5.89	3.05	−0.12 to 11.89	−0.36	1.76	−3.83 to 3.12
Separated/divorced/widowed	6.58	3.31	0.08–13.08*	5.18	1.95	1.34 to 9.01**
**Level of education**						
No formal education	Ref					
Primary education	−1.44	4.00	−9.30 to 6.42			
Secondary education	2.15	4.03	−5.78 to 10.07			
Higher education	1.84	4.86	−7.71 to 11.40			
**Employment situation**						
Active worker	Ref					
Inactive worker	−8.23	4.12	−16.34 to −0.12*			
Disabled	−11.28	3.35	−17.87 to −4.69**			
Others	−6.49	3.91	−14.18 to 1.19			
**People they live with**						
Alone	Ref					
With others	−3.88	2.65	−9.09 to 1.33			
**Evolution of psychotic disorder in years**	−0.07	0.10	−0.27 to 0.13			
**Family history of mental disorders**						
Yes	Ref			Ref		
No	−4.48	2.07	−8.56 to −0.40*	−2.63	1.20	−4.98 to −0.27*
**Number of hospital admissions**	−0.09	0.28	−0.63 to 0.45			
**Medical history**						
Yes	Ref					
No	4.86	2.74	−0.53 to 10.26			
**Number of prescribed antipsychotics**						
1 antipsychotic	Ref					
2 antipsychotics	0.12	2.21	−4.23 to 4.48			
≥ 3 antipsychotics	−13.44	3.41	−20.16 to −6.73***			
**Treatment with anxiolytics**		−1.72				
Yes	Ref					
No	−5.87	2.16	−10.11 to −1.63*			
**Treatment with antidepressants**						
Yes	Ref					
No	−7.48	2.27	−11.95 to −3.01***			
**Suicide attempts**						
Yes	Ref					
No	−9.21	2.36	−13.86 to −4.56***			

*OLS, overall life satisfaction; PMH, positive mental health; PWI-A, Personal Wellbeing Index-Adult; SD, standard deviation; SWB, subjective wellbeing; 95% CI, 95% confidence interval*.

^*^p < 0.05.

^**^p < 0.01.

^***^p < 0.001.

## Discussion

The participants in our study presented lower levels of PMH than sample populations without mental disorders who had responded to the PMHQ (Lluch-Canut et al., 2013; Albacar, 2014; Roldán-Merino et al., 2017; Puig-Llobet et al., 2020; Sequeira et al., 2020). However, results were similar to findings in a study on patients with schizophrenia (Miguel, 2014). These results are a cause for concern and reinforce the importance of developing services and interventions aimed at improving PMH in patients with psychotic disorder.

The level of recovery was similar to that of a sample of patients with severe mental disorder (Drapalski et al., 2016). In contrast, it was lower than in a sample of patients included in an early intervention program (EIP) in psychosis (Bhullar et al., 2018). These results demonstrate the effectiveness of EIP programs in terms of recovery.

The PWI-A score for SWB was low (M = 60.20) compared to the normative range within Western populations, which is between 70 and 80 points (International Wellbeing Group, 2013). One study conducted in Israel on patients with severe mental disorder obtained similar results (Werner, 2012). Cummins et al. (2014) argued that SWB may decrease in response to adverse events, but these deviations are generally temporary thanks to the action of a homeostatic mechanism. However, when normal feelings of wellbeing disappear, they are replaced by depression (Cummins, 2010). The most plausible hypothesis put forward is that psychotic disorder and the life experience that accompanies severe mental illness may affect this population's SWB.

No studies were found in the literature in which OLS had been administered to patients with psychotic disorder. The data we obtained were consistent with the study by Fervaha et al. (2016), which showed that patients with schizophrenia were less happy in general than healthy controls.

The level of insight in the sample was generally low (Birchwood et al., 1994), although 45.3% of the patients presented adequate insight. This finding is consistent with a literature review in which it was estimated that between 50 and 80% of patients with schizophrenia partially, or totally, lacked awareness of their mental illness (Raffard et al., 2008).

The mean GAF score was similar to that of other studies in European populations (Gaite et al., 2005) and in the Spanish population (Al-Halabí et al., 2016). Scientific evidence has shown that diagnosis and an EIP could significantly improve therapeutic results and enhance the effectiveness of established treatments (Arango, 2015).

When analyzing PMH and sociodemographic and clinical variables, some aspects of interest were found that are relevant for discussion. Single patients presented a lower level of global PMH than married, separated or widowed subjects. These data are similar to findings in other studies conducted on patients with mental disorders (Vaingankar et al., 2013; Sambasivam et al., 2016), which used other instruments to measure PMH. In contrast, patients in active employment reported higher global PMH, in line with other research (Sambasivam et al., 2016; Na and Lim, 2020), although the PMHQ was not used.

As in other studies in which the PMHQ was used, with regard to factor F2, women reported having greater prosocial behavior (Lluch-Canut et al., 2013; Albacar, 2014). These results also coincided with other studies conducted on patients with mental disorders (Vaingankar et al., 2013; Sambasivam et al., 2016; Jeyagurunathan et al., 2017). Single patients reported lower prosocial behavior compared to those who were married, separated, divorced or widowed. The data we obtained were consistent with the study by Sambasivam et al. (2016) conducted on patients with mental disorders, although the PMHQ was not used. In contrast, in the study carried out by Miguel (2014), single patients presented greater prosocial behavior. Similarly, patients in active employment reported greater prosocial behavior, whereas patients with some type of disability had the lowest scores. These results are in line with other studies that show lower levels of PMH among the unemployed or people who have unstable jobs (Sambasivam et al., 2016), although the PMHQ was not used.

It was found that patients with the highest score in factor F4 were those who lived alone and those who were separated, divorced or widowed. In contrast, single patients showed more autonomy. These results are consistent with the study by Sambasivam et al. (2016), where single people reported lower levels of autonomy, although the PMHQ was not used.

With regard to factor F5, higher values were observed among patients who were married or active workers. In contrast, single and disabled patients showed a lower capacity for problem-solving and self-actualization. These data are similar to other studies (Vaingankar et al., 2013; Sambasivam et al., 2016) that used other instruments to measure PMH.

Finally, women obtained higher scores in interpersonal relationship skills than men with regard to the F6 factor, coinciding with other studies (Lluch-Canut et al., 2013). However, married patients were found to have greater interpersonal relationship skills. These data are similar to other studies (Sambasivam et al., 2016), although the PMHQ was not used. Patients in active employment reported greater interpersonal relationship skills, coinciding with similar results in other studies (Vaingankar et al., 2013; Sambasivam et al., 2016) in which the PMHQ was not used.

Based on the literature, few research studies have related the level of PMH measured with the PMHQ and the clinical variables used in this study among patients with psychotic disorder. Patients with a family history of mental illness, those prescribed three or more antipsychotics, those being treated with anxiolytics, and also those who had attempted suicide reported lower levels of PMH. Similar data were obtained in the study carried out by Lluch-Canut et al. (2013), which used the PMHQ to show that polymedicated patients presented lower overall PMH levels. These results can be explained by the prevalence of comorbidity of anxiety and depressive disorders in schizophrenia, which is significantly higher than in the general population (Buckley et al., 2009; Kiran and Chaudhury, 2016). Epidemiological studies have shown that polypharmacy is extremely common in schizophrenia, although evidence of its benefits remains scant (Ballon and Stroup, 2013).

When analyzing specific PMH factors and clinical variables, results in the F1 factor showed that patients prescribed three or more antipsychotics, those being treated with antidepressants, and those who had attempted suicide reported lower personal satisfaction, although no studies with similar results were found. In factors F3, F4 and F5, it was observed that patients prescribed three or more antipsychotics, those being treated with antidepressants, and those who had attempted suicide reported less self-control, autonomy and capacity for problem-solving and self-actualization. Similar data were obtained in Lluch-Canut et al. (2013), showing that polymedicated patients presented lower levels in these three factors. Moreover, it was found in factor F3 that patients with a family history of mental illness showed less capacity for self-control. Regarding factor F5, it was observed that patients with fewer years of evolution of the psychotic disorder reported a lower capacity for problem-solving and self-actualization. Finally, regarding factor F6, patients prescribed three or more antipsychotics and those being treated with anxiolytics or antidepressants reported lower interpersonal relationship skills.

Regarding the relationship between PMH and recovery, it was observed that both the global levels of PMH and the PMHQ factors correlated positively with the MARS. One study on patients with mental disorders had similar results, although different instruments were used (Iasiello et al., 2019). The results have indicated that PMH can be an important resource for patients to recover from a mental disorder and stay mentally healthy. Similarly, based on the analysis of the relationship between PMH and SWB, it would appear that global PMH levels and PMHQ factors correlated positively with the PWI-A and the OLS. Several studies can be found with similar results, although different instruments were used (Seow et al., 2016; Vaingankar et al., 2016). Based on these results, it can be asserted that mental health is directly related to SWB.

As for the relationship between PMH and insight, it was observed that both global levels of PMH and PMHQ factors correlated negatively with the overall level and dimensions of insight. However, no studies exist linking these two constructs. Similarly, a relationship was found between terms related to PMH (wellbeing, quality of life, etc.) and insight in patients with psychotic disorder, associating high levels of insight with poorer quality of life and wellbeing (Lien et al., 2018; Davis et al., 2020).

Finally, a positive correlation was obtained between PMH and functioning. Accordingly, several studies on patients with mental disorders have shown a similar association (Seow et al., 2016; Vaingankar et al., 2016), although other measurement instruments were used.

The present study reveals that PMH is significantly positively associated with recovery, SWB, functioning, age and being separated, divorced or widowed. In contrast, insight and having a family history of mental disorder showed a significant and negative association with global PMH. However, according to the literature consulted, no studies are available that have analyzed this set of variables that may favor the level of PMH in patients with psychotic disorder.

Various interventions to improve PMH exist in a variety of modalities, including individual, group and online formats, but targeting the general population (Bolier et al., 2013; Teixeira et al., 2019; Eisenstadt et al., 2021). However, no evidence-based intervention research on PMH in the psychotic disorder population has been found. However, there are possible interventions that could provide substantial benefits to PMH in this population, such as cognitive remediation (Vita et al., 2021), progressive muscle relaxation (Melo-Dias et al., 2019) or social cognition training (d'Arma et al., 2021).

### Limitations

The cross-sectional nature of this study did not allow conclusions to be drawn about the causal relationships between the variables that were shown to be related. In this respect, patient follow-up could be of interest. Moreover, the sample was not randomly selected, but consecutive sampling was used, which is considered the best form of non-probability sampling because all available subjects are included.

## Conclusion

This study contributes to increasing the scant existing knowledge of PMH in patients with psychotic disorder. It also helps us to identify the variables, and the relationships between them, which have the greatest impact on mental health. Accordingly, they can be considered when designing interventions, strategies and programs for this type of population. Specific interventions should be made to improve PMH since levels are lower in patients with psychotic disorder than in the general population.

### Relevance for Clinical Practice

Results regarding the PMH profile of patients with psychotic disorder have shown that it is necessary to take the sociodemographic and clinical characteristics of each individual into account in order to carry out specific interventions to reinforce, enhance or maintain PMH levels.

If actions are taken to increase PMH, recovery, SWB and functioning will also increase. At the same time, interventions should be carried out to boost insight, since increasing PMH could decrease insight in people with psychotic disorder.

These data on the needs of patients with psychotic disorder, based on a positive approach to mental health, can be useful for professional practice. They can help guide programs and interventions carried out in the different mental healthcare facilities.

## Data Availability Statement

The original contributions presented in the study are included in the article/supplementary files, further inquiries can be directed to the corresponding author.

## Ethics Statement

The studies involving human participants were reviewed and approved by the Clinical Research Ethics Committee at the Healthcare Institute (Reference Number: S041-1094), the Benito Menni Mental Healthcare Complex (Reference Number: PR-2015-19), the Mar Health Park (Reference Number: 2015/6303/I), and by the Research Committee at the Sant Joan de Déu Health Park. The patients/participants provided their written informed consent to participate in this study.

## Author Contributions

MB-B, MG-C, and ML-C conceptualized the study. MB-B, MG-C, DJ-C, and ML-C contributed to the preparation of the final draft and study protocols. MB-B collected data, conducted initial data analysis, and wrote the first draft. All authors contributed to the article and approved the submitted version.

## Conflict of Interest

The authors declare that the research was conducted in the absence of any commercial or financial relationships that could be construed as a potential conflict of interest.

## Publisher's Note

All claims expressed in this article are solely those of the authors and do not necessarily represent those of their affiliated organizations, or those of the publisher, the editors and the reviewers. Any product that may be evaluated in this article, or claim that may be made by its manufacturer, is not guaranteed or endorsed by the publisher.

## References

[B1] Albacar N. (2014). Atenció d'infermeria a la cuidadora principal de persones amb esquizofrènia: Valoració dels requisits d'autocura i de la salut mental positiva [Tesis doctoral. Universitat Rovira i Virgili]. Available online at: https://www.tesisenred.net/bitstream/handle/10803/294732/Tesi.pdf?sequence=1andisAllowed=y (accessed December 1, 2021).

[B2] Al-Halabí S. Sáiz P. A. Garrido M. Galván G. Casares M. J. Bobes-Bascarán M. T.. (2016). Psychometric properties of a Spanish-version of the Schizophrenia Objective Functioning Instrument (Sp-SOFI). Int. J. Clin. Health Psychol. 16, 58–75. 10.1016/j.ijchp.2015.07.004 30487851 PMC6225053

[B3] American Psychiatric Association (2002). Manual diagnóstico y estadístico de los trastornos mentales DSM-IV-TR. Washington, DC: Masson, APA.

[B4] American Psychiatric Association (2013). Manual diagnóstico y estadístico de los trastornos mentales DSM-5 (5a ed.). Washington, DC: Editorial Médica Panamericana, APA.

[B5] Arango C. (2015). First-episode psychosis research: time to move forward (by looking backwards). Schizophr. Bull. 41, 1205–1206. 10.1093/schbul/sbv126 26392627 PMC4601723

[B6] Ballon J. Stroup T. S. (2013). Polypharmacy for schizophrenia. Curr. Opin. Psychiatry 26, 208–213. 10.1097/YCO.0b013e32835d9efb 23318662 PMC4026924

[B7] Bhullar G. Norman R. M. G. Klar N. Anderson K. K. (2018). Untreated illness and recovery in clients of an early psychosis intervention program: a 10-year prospective cohort study. Soc. Psychiatry Psychiatr. Epidemiol. 53, 171–182. 10.1007/s00127-017-1464-z 29188310

[B8] Birchwood M. Smith J. Drury V. Healy J. Macmillan F. Slade M. (1994). A self-report Insight Scale for psychosis: reliability, validity and sensitivity to change. Acta Psychiatr. Scand. 89, 62–67. 7908156 10.1111/j.1600-0447.1994.tb01487.x

[B9] Bolier L. Haverman M. Westerhof G. J. Riper H. Smit F. Bohlmeijer E. (2013). Positive psychology interventions: a meta-analysis of randomized controlled studies. BMC Public Health 13, 119. 10.1186/1471-2458-13-119 23390882 PMC3599475

[B10] Buckley P. F. Miller B. J. Lehrer D. S. Castle D. J. (2009). Psychiatric comorbidities and schizophrenia. Schizophr. Bull. 35, 383–402. 10.1093/schbul/sbn135 19011234 PMC2659306

[B11] Burgess P. Pirkis J. Coombs T. Rosen A. (2011). Assessing the value of existing recovery measures for routine use in Australian mental health services. Australian and New Zealand Journal of Psychiatry 45, 267–280. 10.3109/00048674.2010.549996 21314238

[B12] Campbell A. Converse P. E. Rodgers W. L. (1976). The Quality of American Life: Perceptions, Evaluations and Satisfactions. London: Rusell Sage.

[B13] Camprubi N. Almela A. Garre-Olmo J. (2008). Psychometric properties of the Spanish validation of the Insight Scale. Actas Españ. Psiquiatr. 36, 323–330. 18833495

[B14] Cannavò D. Minutolo G. Battaglia E. Aguglia E. (2016). Insight and recovery in schizophrenic patients. Int. J. Psychiatry Clin. Pract. 20, 83–90. 10.3109/13651501.2016.1141960 26902557

[B15] Casas F. (2010). El bienestar personal: su investigación en la infancia y la adolescencia. Encuentr. Psicol. Soc. 5, 85–101.

[B16] Casas F. Coenders G. Cummins R. A. González M. Figuer C. Malo S. (2008). Does subjective well-being show a relationship between parents and their children?. J. Happiness Stud. 9, 197–205. 10.1007/s10902-007-9044-7

[B17] Chan R. C. H. Mak W. W. S. Chio F. H. N. Tong A. C. Y. (2018). Flourishing with psychosis: a prospective examination on the interactions between clinical, functional, and personal recovery processes on well-being among individuals with schizophrenia spectrum disorders. Schizophr. Bull. 44, 778–786. 10.1093/schbul/sbx120 28981851 PMC6007346

[B18] Cummins R. A. (2010). Subjective wellbeing, homeostatically protected mood and depression: a synthesis. J. Happiness Stud. 11, 1–17. 10.1007/s10902-009-9167-0

[B19] Cummins R. A. Eckersley R. Pallant J. van Vugt J. Misajon R. (2003). Developing a national index of subjective wellbeing: The Australian Unity Wellbeing Index. Soc. Indic. Res. 64, 159–190. 10.1023/A:1024704320683

[B20] Cummins R. A. Li N. Wooden M. Stokes M. (2014). A demonstration of set-points for subjective wellbeing. J. Happiness Stud. 15, 183–206. 10.1007/s10902-013-9444-9

[B21] d'Arma A. Isernia S. Di Tella S. Rovaris M. Valle A. Baglio F.. (2021). Social cognition training for enhancing affective and cognitive theory of mind in schizophrenia: a systematic review and a meta-analysis. J. Psychol. 155, 26–58. 10.1080/00223980.2020.1818671 33048659

[B22] David A. S. (1990). Insight and psychosis. Br. J. Psychiatry 156, 798–808. 10.1192/bjp.156.6.798 2207510

[B23] Davis B. J. Lysaker P. H. Salyers M. P. Minor K. S. (2020). The insight paradox in schizophrenia: a meta-analysis of the relationship between clinical insight and quality of life. Schizophr. Res. 2020:S0920996420304035. 10.1016/j.schres.2020.07.017 32763114

[B24] Drapalski A. L. Medoff D. Dixon L. Bellack A. (2016). The reliability and validity of the Maryland Assessment of Recovery in Serious Mental Illness Scale. Psychiatry Res. 239, 259–264. 10.1016/j.psychres.2016.03.031 27039010

[B25] Drapalski A. L. Medoff D. Unick G. J. Velligan D. I. Dixon L. B. Bellack A. S. (2012). Assessing recovery of people with serious mental illness: development of a new scale. Psychiatric Serv. 63, 48–53. 10.1176/appi.ps.201100109 22227759

[B26] Eisenstadt M. Liverpool S. Infanti E. Ciuvat R. M. Carlsson C. (2021). Mobile apps that promote emotion regulation, positive mental health, and well-being in the general population: systematic review and meta-analysis. JMIR Mental Health 8, e31170. 10.2196/31170 34747713 PMC8663676

[B27] Fervaha G. Agid O. Takeuchi H. Foussias G. Remington G. (2016). Life satisfaction and happiness among young adults with schizophrenia. Psychiatry Res. 242, 174–179. 10.1016/j.psychres.2016.05.046 27288735

[B28] Forsman A. K. Wahlbeck K. Aaro L. E. Alonso J. Barry M. M. Brunn M.. (2015). Research priorities for public mental health in Europe: Recommendations of the ROAMER project. Eur. J. Public Health 25, 249–254. 10.1093/eurpub/cku232 25678606

[B29] Gaite L. Vázquez-Barquero J. L. Herrán A. Thornicroft G. Becker T. Sierra-Biddle D.. (2005). Main determinants of Global Assessment of Functioning score in schizophrenia: a European multicenter study. Compr. Psychiatry 46, 440–446. 10.1016/j.comppsych.2005.03.006 16275211

[B30] Iasiello M. van Agteren J. Keyes C. L. M. Cochrane E. M. (2019). Positive mental health as a predictor of recovery from mental illness. J. Affect. Disord. 251, 227–230. 10.1016/j.jad.2019.03.065 30927584 PMC6487880

[B31] International Wellbeing Group (2013). Personal Wellbeing Index (5th Edition). Melbourne, Australian Centre on Quality of Life, Deakin University. Available online at: http://www.acqol.com.au/uploads/pwi-a/pwi-a-english.pdf (accessed November 10, 2021).

[B32] Jahoda M. (1958). Current Concepts of Positive Mental Health. New York, NY: Basic Books.

[B33] Jeyagurunathan A. Vaingankar J. A. Abdin E. Sambasivam R. Seow E. Pang S.. (2017). Gender differences in positive mental health among individuals with schizophrenia. Compr. Psychiatry 74, 88–95. 10.1016/j.comppsych.2017.01.005 28113098

[B34] Keyes C. L. M. (2013). Promoting and protecting positive mental health: early and often throughout the lifespan. In: Keyes CLM editor. Mental Well-Being. Netherlands: Springer. p. 3–28. 10.1007/978-94-007-5195-8_1

[B35] Kiran C. Chaudhury S. (2016). Prevalence of comorbid anxiety disorders in schizophrenia. Ind. Psychiatry J. 25, 35. 10.4103/0972-6748.196045 28163406 PMC5248417

[B36] Lien Y. J. Chang H. A. Kao Y. C. Tzeng N. S. Lu C. W. Loh C. H. (2018). Insight, self-stigma and psychosocial outcomes in Schizophrenia: a structural equation modelling approach. Epidemiol. Psychiatr. Sci. 27, 176–185. 10.1017/S2045796016000950 27974084 PMC6998951

[B37] Lluch-Canut M. T. (1999). Construcción de una escala para evaluar la salud mental positiva [Tesis doctoral. Universidad de Barcelona]. Available online at: http://diposit.ub.edu/dspace/bitstream/2445/42359/1/E_TESIS.pdf (accessed December 1, 2021).

[B38] Lluch-Canut M. T. Puig-Llobet M. Sánchez-Ortega A. Roldán-Merino J. Ferré-Grau C Positive Mental Health Research Group. (2013). Assessing positive mental health in people with chronic physical health problems: correlations with socio-demographic variables and physical health status. BMC Public Health 13, 928. 10.1186/1471-2458-13-928 24093443 PMC3853147

[B39] Melo-Dias C. Lopes R. C. Cardoso D. F. B. Bobrowicz-Campos E. Apóstolo J. L. A. (2019). Schizophrenia and progressive muscle relaxation—a systematic review of effectiveness. Heliyon 5, e01484. 10.1016/j.heliyon.2019.e01484 31049425 PMC6479115

[B40] Miguel M. D. (2014). Valoración de la Salud Mental Positiva y de los Requisitos de Autocuidado, en pacientes hospitalizados diagnosticados de Esquizofrenia, según la Teoría de Enfermería de Dorothea Orem [Tesis doctoral Universidad de Barcelona]. Available online at: http://diposit.ub.edu/dspace/bitstream/2445/56453/1/MDMR_TESIS.pdf (accessed December 1, 2021).

[B41] Na E.-Y. Lim Y.-J. (2020). Influence of employment on the positive mental health of individuals with schizophrenia living in the community. Psychiatric Q. 91, 203–208. 10.1007/s11126-019-09686-5 31811582

[B42] Puig-Llobet M. Sánchez Ortega M. Lluch-Canut M. Moreno-Arroyo M. Hidalgo Blanco M. A. Roldán-Merino J. (2020). Positive mental health and self-care in patients with chronic physical health problems: Implications for evidence-based practice. Worldviews Evid.-Based Nurs. 17, 293–300. 10.1111/wvn.12453 32762130

[B43] Raffard S. Bayard S. Capdevielle D. Garcia F. Boulenger J. P. Gely-Nargeot M. C. (2008). Lack of insight in schizophrenia: a review. Part I: theoretical concept, clinical aspects and Amador's mode. L'Encéphale 34, 597–605. 10.1016/j.encep.2007.10.008 19081457

[B44] Roldán-Merino J. Lluch-Canut M. T. Casas I. Sanromà-Ortíz M. Ferré-Grau C. Sequeira C.. (2017). Reliability and validity of the Positive Mental Health Questionnaire in a sample of Spanish university students. J. Psychiatr. Ment. Health Nurs. 24, 123–133. 10.1111/jpm.12358 28150373

[B45] Sambasivam R. Vaingankar J. A. Chong S. A. Abdin E. Jeyagurunathan A. Seow L. S. E.. (2016). Positive mental health in outpatients: comparison within diagnostic groups. BMC Psychiatry 16, 412. 10.1186/s12888-016-1113-1 27863524 PMC5116138

[B46] Seow L. S. E. Vaingankar J. A. Abdin E. Sambasivam R. Jeyagurunathan A. Pang S.. (2016). Positive mental health in outpatients with affective disorders: Associations with life satisfaction and general functioning. J. Affect. Disord. 190, 499–507. 10.1016/j.jad.2015.10.021 26561940

[B47] Sequeira C. Carvalho J. C. Gonçalves A. Nogueira M. J. Lluch-Canut T. Roldán-Merino J. (2020). Levels of positive mental health in portuguese and spanish nursing students. J. Am. Psychiatr. Nurses Assoc. 26, 483–492. 10.1177/1078390319851569 31122109

[B48] Sequeira C. Carvalho J. C. Sampaio F. Sá L. Lluch-Canut M. T. Roldán-Merino J. (2014). Evaluation of the psychometric properties of the Positive Mental Health Questionnaire in Portuguese higher education students. Rev. Portug. Enfermagem Saúde Mental 11, 45–53.

[B49] Substance Abuse and Mental Health Services Administration (2005). National Consensus Statement on Mental Health Recovery. Rockville, MD: Department of Health and Human Services.

[B50] Teixeira S. M. A. Coelho J. C. F. Sequeira C. A. da C. Lluch i Canut M. T. Ferré-Grau C. (2019). The effectiveness of positive mental health programs in adults: a systematic review. Health Social Care Commun. 27, 1127–1134. 10.1111/hsc.12776 31144395

[B51] Teke C. Baysan Arabaci L. (2018). The validity and reliability of Positive Mental Health Scale. Anadolu Psikiyatri Derg 19, 21–28.

[B52] Vaingankar J. A. Abdin E. Chong S. A. Sambasivam R. Jeyagurunathan A. Seow E.. (2016). Psychometric properties of the positive mental health instrument among people with mental disorders: a cross-sectional study. Health Qual. Life Outcomes 14, 19. 10.1186/s12955-016-0424-8 26868835 PMC4751680

[B53] Vaingankar J. A. Subramaniam M. Abdin E. Picco L. Phua A. Chua B. Y.. (2013). Socio-demographic correlates of positive mental health and differences by depression and anxiety in an Asian community sample. Ann. Acad. Med. Singap. 42, 514–523. 24254238

[B54] Vita A. Barlati S. Ceraso A. Nibbio G. Ariu C. Deste G.. (2021). Effectiveness, core elements, and moderators of response of cognitive remediation for schizophrenia: a systematic review and meta-analysis of randomized clinical trials. JAMA Psychiatry 78, 848–858. 10.1001/jamapsychiatry.2021.0620 33877289 PMC8058696

[B55] Wahlbeck K. (2015). Public mental health: the time is ripe for translation of evidence into practice. World Psychiatry 14, 36–42. 10.1002/wps.20178 25655149 PMC4329888

[B56] Werner S. (2012). Subjective well-being, hope, and needs of individuals with serious mental illness. Psychiatry Res. 196, 214–219. 10.1016/j.psychres.2011.10.012 22382051

[B57] Widschwendter C. G. Kemmler G. Rettenbacher M. A. Yalcin-Siedentopf N. Hofer A. (2018). Subjective well-being, drug attitude, and changes in symptomatology in chronic schizophrenia patients starting treatment with new-generation antipsychotic medication. BMC Psychiatry 18, 212. 10.1186/s12888-018-1791-y 29954366 PMC6022409

[B58] World Health Organization (2001). International Classification of Functioning, Disability and Health (ICF). Available online at: http://apps.who.int/iris/bitstream/handle/10665/42407/9241545429.pdf?sequence=1 (accessed November 10, 2021).

[B59] World Health Organization (2005). Promoting Mental Health: Concepts, Emerging Evidence, Practice. Available online at: https://apps.who.int/iris/bitstream/handle/10665/42940/9241591595.pdf (accessed November 10, 2021).

[B60] Yu Y. Xiao X. Yang M. Ge X. Li T. Cao G.. (2020). Personal recovery and its determinants among people living with schizophrenia in China. Front. Psychiatry 11, 602524. 10.3389/fpsyt.2020.602524 33362611 PMC7759546

